# Rhazes on the Rejection of "Emission Theory" of Vision

**DOI:** 10.34172/aim.31205

**Published:** 2025-03-01

**Authors:** Mojtaba Heydari, Mohammad Reza Talebnejad, Narges Tajik

**Affiliations:** ^1^Poostchi Ophthalmology Research Center, Department of Ophthalmology, School of Medicine, Shiraz University of Medical Sciences, Shiraz, Iran; ^2^Department of History of Medicine, School of Persian Medicine, Tehran University of Medical Sciences Tehran, Iran; ^3^Scientific Student Association of History of Medicine, Pharmacy and Veterinary, Student Scientific Research Center, Tehran University of Medical Sciences, Tehran, Iran

## Dear Editor,

 Vision, the intricate process of perceiving the external world through the eyes, has long captivated the interest of medical and philosophical minds.^1^ Throughout the annals of history, diverse theories have sought to elucidate the underlying mechanisms governing this complex phenomenon. Among the earliest contenders were the ancient Greeks and Byzantines, who posited theories of vision grounded in “*emission”* or “*extramission ”* hypotheses.^2^ Emission theory, championed by scientists such as Plato and Euclid, postulated that vision transpires through the emission of rays from the eyes.^3^ Euclid’s seminal works, “Optics” and “Catoptrics,” laid the foundational principles for understanding optical properties, while Ptolemy’s “Optics” further expanded upon theories of direct vision, reflection, and refraction.^4^ Galen, the eminent physician of the 2nd century, endorsed emission theory in his seminal treatise “De Usu Partium Corporis Humani” wielding significant influence over medical and philosophical thought of his era.^5^

**Figure 1:**
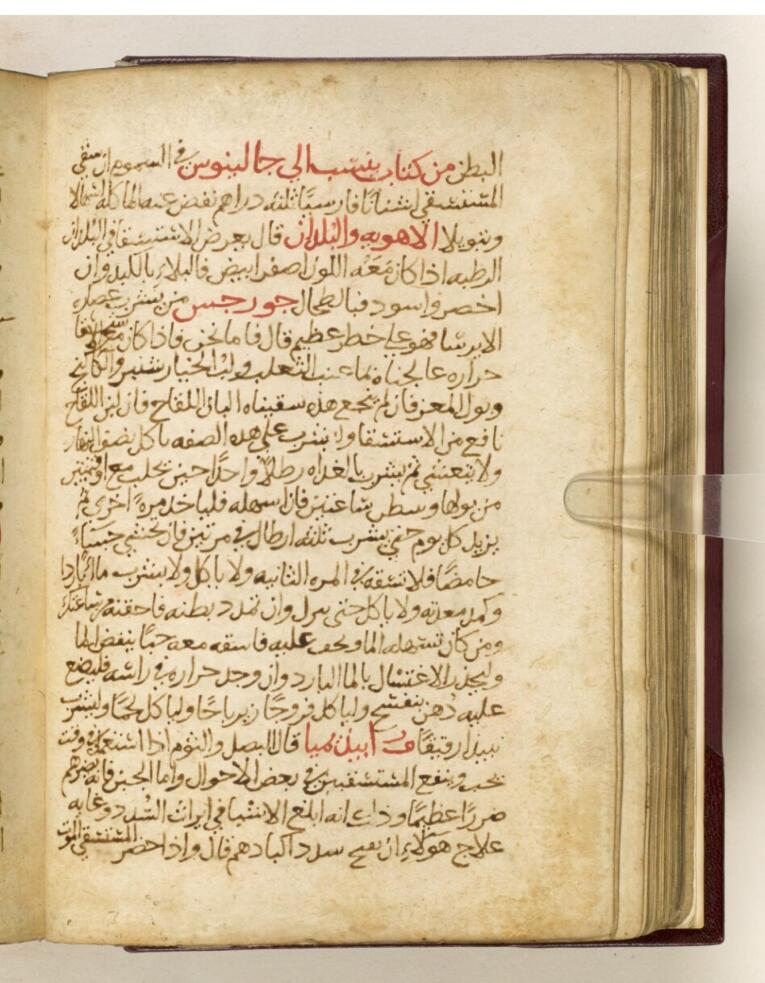
A Page from the Manuscript "*Al- Havi*" by Rhazes. In this book Rhazes quoted and criticized Galen’s medical theories in multiple parts

 However, with the emergence of the Islamic golden age, Rhazes (865‒925 CE),^6^ a towering figure in the field of medicine, marked a pivotal change in the discourse on vision. Born in Rey, Iran, Rhazes challenged the prevailing orthodoxy of Galen’s emission theory in his seminal work, “Doubts on Galen”^7^ (Figure 1). In this groundbreaking treatise, Rhazes embarked on a comprehensive critique of Galen’s theories, spanning twenty chapters. The fourth chapter dedicated to dismantling the pillars of emission theory. One of Rhazes’ primary objections lay in Galen’s explanations for phenomena such as the brightness of animal eyes in darkness and the dilation of the pupil in response to eye closure. Instead of attributing these observations to emitted rays, Rhazes proposed alternative explanations grounded in principles of reflection and light adjustment. Moreover, Rhazes contested Galen’s notion of a hole in the optic nerve as a conduit for emitted light, positing instead a theory centered on the transmission of the visionary soul to the brain. This rejection of extramission theory represented a paradigm shift in the understanding of vision, challenging centuries-old dogma with empirical observation and rational inquiry.

 Rhazes’ critique reverberated through the intellectual landscape of the Middle Ages, influencing subsequent Muslim scholars such as Alhazen and Avicenna, who embraced his intromission theory of vision.^5^ By prioritizing empirical evidence over entrenched belief systems, Rhazes paved the way for a more nuanced understanding of vision—one that continues to shape scientific inquiry to this day. Rhazes’ “Doubts on Galen” stands as a testament to the power of skepticism and critical thinking in advancing our understanding of the natural world. His rejection of emission theory represents a milestone in the history of optics, underscoring the importance of questioning established paradigms and embracing a spirit of inquiry in the pursuit of knowledge.
